# Serum Fat-Soluble Vitamin Levels of 6,082 Minors in Zhuzhou City

**DOI:** 10.1155/2022/4673964

**Published:** 2022-12-01

**Authors:** Qiong Tang, Xiao-Min Ye, Yi-Can Yang, Shi-Bin Zhang, Li-Juan Yan, Dai Gong, Li Zou, Xiang-Lan Wen

**Affiliations:** ^1^Department of Children Health Care Center, Zhuzhou Hospital Affiliated to Xiangya Medical College, Central South University, Zhuzhou, Hunan 412007, China; ^2^Department of Pediatrics, Zhuzhou Hospital Affiliated to Xiangya Medical College, Central South University, Zhuzhou, Hunan 412007, China

## Abstract

**Objective:**

To explore the nutritional status of serum fat-soluble vitamins such as vitamin A, 25-hydroxyvitamin D, and vitamin E of minors in the Zhuzhou area to provide a scientific basis for clinical guidance to supplement fat-soluble vitamins reasonably.

**Method:**

A total of 6,082 minors who underwent physical examination from January 2017 to February 2019 in the Children's Health Department of Zhuzhou Hospital affiliated with XiangYa School of Medicine of Central South University were selected as the subjects to measure the levels of serum fat-soluble vitamins A, D, and E.

**Results:**

(1) Their average levels of serum vitamin A, 25-hydroxyvitamin D, and vitamin E were (0.34 ± 0.08) mg/mL, (34.65 ± 10.24) ng/mL, and (10.11 ± 2.65) mg/mL, respectively. (2) Serum vitamin E showed a gender difference (*P* < 0.001). (3) The average levels of serum 25-hydroxyvitamin D and vitamin E in infancy, early childhood, preschool age, school age, and adolescence decreased gradually (*P* < 0.05). In contrast, the average level of serum vitamin A ranged between 0.32 mg/mL and 0.37 mg/mL. (4) The age was negatively correlated with serum 25-hydroxyvitamin D (*r* = −0.517, *P* < 0.001) and weakly negatively correlated with vitamin E (*r* = −0.366, *P* < 0.001), but weakly positively correlated with vitamin A (*r* = 0.269, *P* < 0.001).

**Conclusion:**

Minors from infancy to adolescence in Zhuzhou should strengthen their supplementation of fat-soluble vitamins.

## 1. Introduction

Serum fat-soluble vitamins are essential nutrients for the human body, playing a vital role in physiology and pathology [1]. Vitamin A plays an important role in the development and function of vision, gonadal function, asthma, childhood pneumonia, and chronic graft-versus-host disease to improve skin fibrosis [1]. Vitamin A can promote the formation of columnar epithelial cells in damaged tissues and regulate the expression of mucin by regulating epidermal growth factors, thus indirectly protecting the airway. Its normal range in children is 0.3–2.25 mg/mL (0.52–1.56 *μ*mol/L). Vitamin A deficiency in children is associated with night blindness xerostomia, corneal softening, corneal ulceration, immune dysfunction, increased risk of infection, susceptibility to respiratory diseases, increased risk of infectious diseases such as measles, and increased risk of death from infectious diseases [2–8].

Vitamin D, also known as antirickets vitamin or calciferol, is an important fat-soluble vitamin, mainly found in the forms of vitamin D (ergocalciferol) and vitamin D3 (cholecalciferol) [2]. It not only prevents rickets but also assists in the treatment of asthma, severe pneumonia, type 2 diabetes, preeclampsia, and other diseases [2]. Vitamin D enhances the absorption of calcium and phosphorus by the body, increases the absorption of phosphorus through the intestinal wall, and increases the reabsorption of phosphorus through the renal tubules, preventing amino acid loss through the kidney [2]. Vitamin D is a steroid derivative and has five compound forms. Among these, vitamin D and vitamin D3 are closely related to health. The active form of vitamin D is 1,25-dihydroxyvitamin D, but due to its short half-life, the level of vitamin D is mainly expressed as 25-hydroxyvitamin D. An amount of 25-hydroxyvitamin D less than 10 ng/mL (25 nmol/l) is defined as a severe deficiency. Less than 20 ng/mL (50 nmol/l) is defined as a deficiency, and less than 30 ng/mL (75 nmol/l) is defined as vitamin D insufficiency. The role of vitamin D in the human body can be divided into classical and nonclassical roles. The classical role of vitamin D is to promote the absorption of calcium and phosphorus by the human intestines and the reabsorption of calcium by renal tubules [9]. The nonclassical role includes the regulation of cell proliferation, differentiation, and function of various tissues in the body [9]. The main symptoms of vitamin D deficiency in children are increased nerve excitability, electrolyte metabolism disorders and skeletal changes, and the harm mainly includes rickets, convulsions, and anemia [9].

Vitamin E, as an antioxidant, plays a vital function in anticoagulation, immunity, metabolism, and fertility [3]. A vitamin E deficiency can lead to infection and ovarian hormone deficiency [3]. The hydrolysate of vitamin E is tocopherol, an antioxidant that aids in scavenging free radicals, resisting oxidation, and promoting protein synthesis and hemoglobin metabolism [10]. Its normal range is 4.9–19.9 mg/mL (11.6–46.4 *μ*mol/L). Studies have shown that vitamin E deficiency during pregnancy can lead to premature delivery and fetal growth restriction and increase the risks of miscarriage, hypertensive disorder complicating pregnancy, gestational diabetes, and other diseases [11]. An insufficient vitamin E level may increase the risk of suffering from chronic diseases such as infection, anemia, and low learning performance, and vitamin E deficiency has adverse effects on children's cognitive ability and motor development [12–14].

The study aims to explore the nutritional status of serum fat-soluble vitamin A, 25-hydroxyvitamin D, and vitamin E of minors in the Zhuzhou area to provide a scientific basis for clinical guidance to supplement fat-soluble vitamins reasonably.

## 2. Subjects and Methods

### 2.1. Subjects

From January 2017 to February 2019, 6,082 minors who underwent physical examination in the Children's Health Department of Zhuzhou Hospital affiliated with XiangYa School of Medicine of Central South University were selected as the subjects. The included subjects were all the children who came to our department for health examination, and they were either healthy or diseased. There were no exclusion criteria in this study. All the parents of children participating in the study were informed of and agreed to the content of the study.

### 2.2. Methods

Collection and grouping of serum fat-soluble vitamin A, 25-hydroxyvitamin D, and vitamin E was done—1.5 mL of venous blood from the subject was collected and stored in a refrigerator at 4°C. Blood samples without anticoagulant were collected and centrifuged directly. The serum was separated as a specimen and sent to Guangzhou Jinyu Medical Inspection Group Co., Ltd. for detection using high-performance liquid chromatography-tandem mass spectrometry (Instrument: AB 4500MD). All specimens were tested within one week by testers specially trained in strict accordance with the instructions for the instruments and reagents. Vitamin A was divided into two groups based on the determination criteria of serum vitamin A nutrition [1], with the concentration of less than 0.30 mg/mL being the vitamin A deficiency group and greater than 0.30 mg/mL being the vitamin sufficiency group. Serum 25-hydroxyvitamin D levels were divided into two groups according to the serum vitamin D nutrition criteria [1], with the concentration of less than 30 ng/mL being the vitamin D deficiency group and greater than or equal to 30 ng/mL being the vitamin D sufficiency group. The normal range of vitamin E is 4.9–19.9 mg/mL (11.6–46.4 *μ*mol/L), so the concentration of less than 4.9 ng/mL is the vitamin E deficiency group and greater than or equal to 4.9 ng/mL is the vitamin E sufficiency group.

All the minors in this study were divided into four groups according to their age: infant group (0–1 year), early childhood group (1–3 years), preschool age (3–6 years), and school age and adolescence (over 6 years). The medical staff participating in this study were all uniformly trained in conducting a questionnaire survey (which include questions of gender and age) on the basic situation of children and the methods of venous blood collection.

### 2.3. Statistical Method

The mean ± standard deviation ($\bar{x}$ ± *s*) was used to describe the measurement data, and the Kolmogorov–Smirnov test was used to analyze the measurement data between two or more groups that do not conform to the normal distribution. The *X*^2^ test was used to analyze the intergroup distribution of enumeration data. Nonparametric correlation analysis was used to analyze the correlation between variables. Statistical analysis was performed by using SPSS 22.0 software package and tested by a two-sided test with the test level *α* = 0.05.

## 3. Results

### 3.1. Basic Situation of Average Level and Data of Serum Fat-Soluble Vitamins of Minors in Zhuzhou

The study investigated 6,082 minors in Zhuzhou city, including 3,524 males and 2,978 females, 3,975 infants, 1,537 young children, 623 preschool age, and 367 school age and adolescence children. Mean serum levels of vitamin A, 25-hydroxyvitamin D, and vitamin E were (0.34 ± 0.08) mg/mL, (34.65 ± 10.24) ng/mL, and (10.11 ± 2.65) mg/mL, respectively.

### 3.2. Gender Distribution and Difference in Serum Fat-Soluble Vitamins among Minors in Zhuzhou

The results showed that there was no gender difference in the average levels of serum vitamin A and 25-hydroxyvitamin D (*P* > 0.05), but there was a difference in the average level of serum vitamin E (*P* < 0.001). The results are reported in Table 1.

### 3.3. Distribution and Difference of Serum Fat-Soluble Vitamins among Minors of Different Age Groups in Zhuzhou

The results indicated differences in the average levels of serum fat-soluble vitamins in different age groups in Zhuzhou. The average levels of 25-hydroxyvitamin D and vitamin E decreased with age. There were significant statistical differences in the average levels of 25-hydroxyvitamin D in different age groups (*P* < 0.001). The results are shown in Table 2.

### 3.4. Distribution and Difference of Serum Fat-Soluble Vitamin Sufficiency among Minors of Different Gender Groups in Zhuzhou

There was no difference between genders in the proportion distribution of serum vitamin A, 25-hydroxyvitamin D, and vitamin E sufficiency (*P*=0.348, *P*=0.943, *P*=0.277). The results are reported in Table 3.

### 3.5. Distribution and Difference of Serum Fat-Soluble Vitamin Sufficiency among Minors of Different Age Groups in Zhuzhou

The proportion of serum 25-hydroxyvitamin D sufficiency was 81.9% in infancy, 56.2% in early childhood, 23.9% in preschool age, and 15.5% in school ages and adolescence, respectively, displaying significant statistical differences among different age stages (*X*^*2*^ = 1432.609, *P* < 0.05). The results are shown in Table 4. However, the proportion of serum vitamin A and vitamin E sufficiency showed no obvious change, and the results are shown in Table 5.

### 3.6. Correlation between Serum Fat-Soluble Vitamins and Age and Genders of Minors in Zhuzhou

The age of 6,082 minors in Zhuzhou was negatively correlated with serum 25-hydroxyvitamin D (*r* = −0.517, *P* < 0.001) and weakly negatively correlated with vitamin E (*r* = −0.366, *P* < 0.001) but weakly positively correlated with vitamin A (*r* = 0.269, *P* < 0.001). There was no correlation between gender and serum fat-soluble vitamins (*P* > 0.05).

## 4. Discussion

Human serum fat-soluble vitamins mainly contain vitamin A, 25-hydroxyvitamin D, and vitamin E, which have essential roles in the human body. Fat-soluble vitamins are indispensable in many physiological processes such as vision, bone health, immune function, weight, and blood coagulation [2]. The research shows that healthy children are deficient in fat-soluble vitamins A, vitamin D, and E to some extent. The Chinese Nutrition Society defined serum vitamin A concentration of less than 0.30 mg/mL as vitamin A deficiency and more than 0.30 mg/mL as vitamin A sufficiency. This study found that although the average level of vitamin A was relatively sufficient in the Zhuzhou area, the proportion of vitamin A sufficiency remains a concern, especially a vitamin A sufficiency in infancy. This suggests that parents should pay special attention to vitamin A supplementation, and medical staff should reinforce popular science knowledge about vitamin A to reduce the chance of children suffering from an infection, and it is recommended that vitamin A should be supplemented for children in China. Vitamin D and parathyroid hormone (PTH) work synergically in assuring bone homeostasis. Since it is well known that hypovitaminosis D and secondary hyperparathyroidism are detrimental for bone health [2]. Vitamin D enhances the absorption of calcium and phosphorus by the body, increases the absorption of phosphorus through the intestinal wall, and increases the reabsorption of phosphorus through the renal tubules, preventing amino acid loss through the kidney [2]. In addition, vitamin D deficiency can lead to rickets, osteoporosis, and other diseases [15, 16]. More studies show that vitamin D deficiency is closely related to cardiovascular diseases, respiratory diseases, diabetes, and immune system diseases [17, 18]. Therefore, it is of great clinical significance to regularly monitor vitamin D concentration in the human body. Many factors influence the level of vitamin D in the human body, such as age, gender, dietary habits, regions, seasons, and diseases. Therefore, doctors should clinically evaluate the level of vitamin D from many aspects. The levels of vitamin D in different regions of China vary. For example, Hanmin et al. found that 97.7% of Shanghai residents' vitamin D concentration was less than 30 ng/mL in autumn and winter. Lichun et al. studied the vitamin D level of people in the Jiaxing area and found that only 4.42% of the population had a vitamin D concentration greater than 30 ng/mL, and the concentration of 25-hydroxyvitamin D changed with the seasons. It increased in summer and autumn and was lower in winter and spring, and the difference was statistically significant. This study found that the level of 25-hydroxyvitamin D decreased with age, which may correlate with parents' lack of awareness of vitamin D deficiency of older children in Zhuzhou and the decrease in annual exposure to sunlight and air pollution. Parents do not appear to understand that their children might suffer from vitamin D deficiency and that they need vitamin D supplementation as they get older. In addition, children's academic pressure also increases with age, and their daily exposure to sunlight decreases. Some researchers and clinicians agree that everyone is at risk of poor vitamin D status. Only few natural foods contain vitamin D; therefore, trying to improve the amount of vitamin D through food is difficult. At the same time, due to insufficient sunlight exposure, the skin will not produce enough vitamin D. Therefore, it is recommended that vitamin D should also be supplemented for children in China. Currently, higher vitamin E levels are found in most neonates, which may be related to higher hormone levels in mothers. However, as children age, they may develop a vitamin E deficiency. The study found that although vitamin E levels in minors of all ages gradually decreased with age, the overall level was relatively adequate. To prevent vitamin E deficiency, clinicians should encourage parents to feed their children butter, egg yolk, and plant seeds; however, poor cooking methods will destroy the structure of vitamin E. In addition, vitamin E is higher in female than male, which could be due to its important role in the organs and metabolism of females.

In general, the level of serum fat-soluble vitamins in Zhuzhou in this study is not optimistic. Clinicians should take active measures to improve children's vitamin status. The average level of vitamin A has just reached the sufficiency level stipulated by the Chinese Nutrition Society; however, the proportion of vitamin A sufficiency in all ages are not high. The average level of 25-hydroxyvitamin D is also generally low. Most of the population in this study are minors currently living in Zhuzhou city, a relatively developed economy. Therefore, considering the generally low level of vitamin D in China's population, it can be deduced that the vitamin D level of minors is insufficient if the scope is extended to the whole Zhuzhou area. This provides a definite clinical basis for clinicians to guide minors in the Zhuzhou area to supplement vitamin D. At the same time, medical staff should increase health education knowledge and inform parents to increase their children's outdoor activities and light time, adjust dietary structures, and improve the intake of vitamin D and calcium. As children age, medical staff and parents should pay attention to their vitamin E levels and monitor them regularly to detect and prevent vitamin E deficiency. It is suggested that minors in Zhuzhou should strengthen the supplementation of fat-soluble vitamins from infancy to adolescence.

## Figures and Tables

**Table 1:** Gender differences in the distribution of serum fat-soluble vitamins in Zhuzhou area.

	Male (median (Q1, Q3))	Female (median (Q1, Q3))	*P*
Vitamin A (mg/ml)	0.34 (0.28, 0.39)	0.34 (0.28, 0.4)	>0.05
25-Hydroxyvitamin D (ng/ml)	33.8 (27.4, 41)	34 (28, 41.1)	>0.05
Vitamin E (mg/ml)^a^	9.4 (7.9, 11.2)	10.2 (8.6, 12.1)	<0.001

**Table 2:** Age differences in the distribution of serum fat-soluble vitamins in Zhuzhou area.

	Vitamin A (mg/ml) (median (Q1, Q3))	25-Hydroxyvitamin D (ng/ml) (median (Q1, Q3))	Vitamin E (mg/ml) (median (Q1, Q3))
Infancy	0.32 (0.27, 0.38)	37.7 (31.8, 44.1)	10.6 (9, 12.4)
Early childhood	0.37 (0.32, 0.43)	31 (26.4, 36.1)	9.1 (7.9, 10.6)
Preschool age	0.35 (0.3, 0.4)	24.8 (21.25, 29.7)	8.2 (7.1, 9.5)
School age and adolescence	0.35 (0.31, 0.4)	22.1 (18.1, 26.75)	7.8 (6.9, 9.1)
*P*	>0.05	<0.001	>0.05

**Table 3:** Gender differences in the distribution of serum fat-soluble vitamins in Zhuzhou area.

	Vitamin A		25-Hydroxyvitamin D		Vitamin E	
	Deficiency	Sufficiency	Deficiency	Sufficiency	Deficiency	Sufficiency
Male	1103 (31.3%)	2421 (68.7%)	1178 (33.4%)	2346 (66.6%)	0 (0%)	3524 (100.0%)
Female	900 (30.2%)	2078 (69.8%)	998 (33.5%)	1980 (66.5%)	1 (0%)	2977 (100.0%)
*P*	0.348		0.943		0.277	

**Table 4:** Distribution difference of 25-hydroxyvitamin D sufficiency in Zhuzhou area.

	25-Hydroxyvitamin D	
	Deficiency	Sufficiency
Infancy	719 (18.1%)	3256 (81.9%)
Early childhood	673 (43.8%)	864 (56.2%)
Preschool age	474 (76.1%)	149 (23.9%)
School age and adolescence	310 (84.5%)	57 (15.5%)
*P*		0.05

**Table 5:** Distribution difference of sufficient vitamin A and E sufficiency in Zhuzhou area.

	Vitamin A		Vitamin E	
	Deficiency	Sufficiency	Deficiency	Sufficiency
Infancy	1541 (38.8%)	2434 (61.2%)	0 (0%)	3975 (100.0%)
Early childhood	242 (15.7%)	1295 (84.3%)	1 (0.1%)	1536 (99.9%)
Preschool age	147 (23.6%)	476 (76.4%)	0 (0%)	623 (100.0%)
School age and adolescence	73 (19.9%)	2940(80.1%)	0 (0%)	367 (100.0%)
*P*		>0.05		>0.05

## Data Availability

The data used to support the findings of this study are available from the corresponding author upon request.

## References

[1] Wopereis S., Booth S. L., Reddy P. (2022). Biochemistry, fat soluble vitamins. *Current Developement Nutrition*.

[2] Sainaghi P. P., Bellan M., Antonini G., Bellomo G., Pirisi M. (2011). Unsuppressed parathyroid hormone in patients with autoimmune/inflammatory rheumatic diseases: implications for vitamin D supplementation. *Rheumatology*.

[3] Hahn H. J., Jung H. J., Schrammek-Drusios M. C. (2016). Instrumental evaluation of anti-aging effects of cosmetic formulations containing palmitoyl peptides, Silybum marianum seed oil, vitamin E and other functional ingredients on aged human skin. *Experimental and Therapeutic Medicine*.

[4] Yee M. M. F., Chin K. Y., Ima-Nirwana S., Wong S. K. (2021). Vitamin A and bone health: a review on current evidence. *Molecules*.

[5] Hod M., Kapur A., Mcintyre H. D. (2019). Evidence in support of the International Association of Diabetes in Pregnancy study groups’ criteria for diagnosing gestational diabetes mellitus worldwide in 2019. *American Journal of Obstetrics and Gynecology*.

[6] Juan J., Yang H. (2020). Prevalence, prevention, and lifestyle intervention of gestational diabetes mellitus in China. *International Journal of Environmental Research and Public Health*.

[7] Chai X. L., Zhang Z. Q., Chen A. P. (2021). Exploring the pharmacological mechanism of danhe granulesagainst hyperlipidemia by means of network pharmacology and verified by preliminary experiments. *World J Tradit Chin Med*.

[8] Chai X. L., Pan Q., Zhang Z. Q., Tian C. Y., Yu T., Yang R. (2021). Effect and signaling pathways of Nelumbinis folium in the treatment of hyperlipidemia assessed by network pharmacology. *World J Tradit Chin Med*.

[9] Zhang H., Liu Y., Fang X. (2021). Vitamin D(3) protects mice from diquat-induced oxidative stress through the NF-*κ*B/Nrf2/HO-1 signaling pathway. *Oxidative Medicine and Cellular Longevity*.

[10] Gugliandolo A., Bramanti P., Mazzon E. (2017). Role of vitamin E in the treatment of alzheimer’s disease: evidence from animal models. *International Journal of Molecular Sciences*.

[11] Ma H., Qiao Z., Li N., Zhao Y., Zhang S. (2021). The relationship between changes in vitamin A, vitamin E, and oxidative stress levels, and pregnancy outcomes in patients with gestational diabetes mellitus. *Annals of Palliative Medicine*.

[12] Wan Z., Wang L., Xu Y. (2021). Hidden hunger of vitamin E among healthy College students: a cross- sectional study. *Endocrine, Metabolic & Immune Disorders - Drug Targets*.

[13] Alghadir A. H., Gabr S. A., Iqbal Z. A., Al-Eisa E. (2019). Association of physical activity, vitamin E levels, and total antioxidant capacity with academic performance and executive functions of adolescents. *BMC Pediatrics*.

[14] Okebukola P. O., Kansra S., Barrett J. (2020). Vitamin E supplementation in people with cystic fibrosis. *Nutrients*.

[15] Adhikari R., White D., House J. D., Kim W. (2020). Effects of additional dosage of vitamin D3, vitamin D2, and 25-hydroxyvitamin D3 on calcium and phosphorus utilization, egg quality and bone mineralization in laying hens. *Poultry Science*.

[16] Ao T., Kikuta J., Ishii M. (2021). The effects of vitamin D on immune system and inflammatory diseases. *Biomolecules*.

[17] Wei Z., Yoshihara E., He N. (2018). Vitamin D switches BAF complexes to protect beta cells. *Cell*.

[18] Zhang S., Miller D. D., Li W. (2021). Non-musculoskeletal benefits of vitamin D beyond the musculoskeletal system. *International Journal of Molecular Sciences*.

